# Macrophages as key modulators of calcific aortic valve disease

**DOI:** 10.3389/fcvm.2025.1664067

**Published:** 2025-09-24

**Authors:** Nervana Issa, Gérémy Blot, Alexandre Candellier, Cédric Boudot, Loïc Louvet, Saïd Kamel, Youssef Bennis, Lucie Hénaut

**Affiliations:** ^1^ UR UPJV 7517, MP3CV, CURS, Amiens, France; ^2^ Department of Biochemistry and Endocrine Biology, Amiens University Hospital, Amiens, France; ^3^ Department of Pharmacology, Amiens University Hospital, Amiens, France

**Keywords:** aortic valve, calcific aortic valve disease, macrophages, inflammation, fibrosis, calcification

## Abstract

Calcific aortic valve disease (CAVD), defined by thickening, fibrosis, and mineralization of the aortic valve (AV) leaflets, is the most common valvular heart disease worldwide. This progressive remodeling gradually impairs valve opening, obstructing blood flow. Without intervention, the resulting aortic stenosis (AS) causes hemodynamic deterioration that ultimately leads to heart failure and death. To date, therapeutic options remain limited, making valve replacement the reference treatment. While valvular endothelial and interstitial cells have traditionally been considered the primary drivers of the osteogenic program underlying AV remodeling, recent evidence highlights a central role for macrophages, whose plasticity profoundly impacts the local microenvironment. In their inflammatory state, macrophages release cytokines, generate oxidative stress, and secrete Bone Morphogenetic Protein 2 (BMP2), which promotes the osteogenic transformation of valvular cells. The resulting calcium crystal deposition further amplifies macrophage-driven inflammation, creating a vicious cycle. Conversely, immunomodulatory macrophages can protect against CAVD by releasing pyrophosphate, a calcification inhibitor. However, these macrophages also secrete pro-fibrotic factors and may undergo myeloid-to-mesenchymal transition, processes that paradoxically contribute to AV fibrosis and mineralization. In addition, macrophages within the AV can differentiate into osteoclast-like cells, suggesting that a bone-like remodeling process occurs in the cardiovascular wall. This high phenotypic plasticity complicates our understanding of CAVD pathogenesis and highlights the need for deeper insight into macrophage functions to design effective preventive and therapeutic strategies. This review summarizes the mechanisms through which different macrophage subsets promote, prevent, or reverse AV remodeling, in both native and bioprosthetic contexts, and explores the therapeutic potential of targeting macrophages or their activity to slow AS progression.

## 1 Introduction

Calcific aortic valve disease (CAVD) is the most prevalent valvular heart disease worldwide, characterized by progressive fibro-calcific remodeling of the aortic valve (AV) leaflets, leading to leaflet thickening and impaired mobility. Over time, this process results in progressive narrowing of the AV opening, known as aortic stenosis (AS), which obstructs blood flow across the valve. When hemodynamically significant, AS increases left ventricular afterload, potentially causing left ventricular hypertrophy (LVH) and heart failure (HF). CAVD affects up to 5% of individuals over 65 and ranks as the third most frequent cardiovascular disease, after hypertension and coronary artery disease (1, 2). With an aging population, its prevalence is expected to double over the next 20 years, amplifying the socioeconomic burden (3). The disease progresses through a long latent phase marked by subtle molecular, cellular, and tissue-level changes that precede clinical symptoms. Because CAVD is typically diagnosed once hemodynamic impairment becomes apparent, opportunities for early therapeutic intervention are currently limited. Untreated, symptomatic CAVD carries a poor prognosis, and no pharmacological therapy has demonstrated efficacy in slowing disease progression. Consequently, aortic valve replacement (AVR)—either surgical (SAVR) or transcatheter (TAVR)— remains the only effective treatment. Improved understanding of early mechanisms may facilitate the identification of novel biomarkers and preventive strategies

A growing body of evidence implicates inflammation as a central driver of CAVD pathogenesis. While elevated circulating levels of inflammatory biomarkers such as TNF*α*, IL-8, and IL-6 have been linked with disease development and prognosis (4, 5), increasing attention is now focused on local inflammatory processes within the AV tissue that drive leaflet remodeling. Among immune cells, macrophages have emerged as key players. Present even in healthy valves as part of immune surveillance, they contribute both to the initiation and resolution of sterile inflammation. Notably, their remarkable plasticity allows them to secrete pro- or anti-inflammatory mediators, as well as regulators of fibrosis and calcification, making them critical modulators of valvular remodeling.

This review summarizes current knowledge on how distinct macrophage subsets promote, prevent, or reverse AV remodeling in both native and bioprosthetic contexts. It also discusses the therapeutic potential of targeting macrophages or their activity to prevent or slow the progression of AS.

## 2 Structure and function of the AV

The human AV is an avascular structure composed of three individual leaflets anchored to a fibrous ring at the outflow of the left ventricle. Each leaflet is composed of three distinct layers of extracellular matrix (ECM), named fibrosa, spongiosa, and ventricularis, lined on both sides by valvular endothelial cells (VECs) (6). The fibrosa, located on the aortic side, consists of dense type I and III collagen arranged circumferentially (7). On the ventricular side, the ventricularis contains radially aligned collagen and elastin fibers (8). Between these, the spongiosa is characterized by a high content of glycosaminoglycans. These ECM layers are mainly populated by valvular interstitial cells (VICs), but also contain fibroblasts, mesenchymal stem cells (9), and a minority of vascular smooth muscle cells (VSMCs, <5%), particularly near the ventricularis base (10, 11). VICs largely derive from endothelial-to-mesenchymal transition (EndMT) (12–15), but are also replenished by hematopoietic stem cells (16). These cells are highly plastic and can differentiate into myofibroblast-, chondrocyte-, osteoblast-, or adipocyte-like phenotypes in response to environmental stimuli, positioning them as central regulators of both valve physiology and pathological remodeling (17). In healthy valves, VICs typically display a quiescent fibroblast-like phenotype (qVICs), and are primarily involved in ECM turnover and collagen homeostasis.

## 3 Main processes involved in AV remodelling

CAVD is an active, tightly regulated cellular process that evolves through two interconnected phases: an initiation phase, marked by endothelial injury and dysfunction that trigger local inflammation, and a progression phase, in which sustained inflammation drives fibrosis and mineralization (18, 19).

### 3.1 Initiation phase

Mechanical stress during the cardiac cycle can injure VECs, disrupting the endothelium and basement membrane. This facilitates the entry of circulating components such as lipids and red blood cells (RBCs) and induces VECs expression of adhesion molecules including E-selectin, VCAM-1, and ICAM-1, which promote immune cell adhesion, rolling, and transmigration into the subvalvular tissue (20–22). Infiltrating monocytes and lymphocytes differentiate into macrophages and activated *T* cells, releasing pro-inflammatory cytokines such as TGF-β, IL-1β, IL-6, and TNF-α. Exposure to RBCs stimulates VICs to produce inflammatory mediators such as IL-6 and IL-1β, amplifying local inflammation. Inflammation rapidly becomes a central feature of CAVD, with immune cell density often correlating with disease severity and tissue remodeling.

### 3.2 Main mechanism driving fibrosis

Fibrosis, which results from excessive ECM production, particularly of collagen (23), is primarily mediated by myofibroblasts. Within the AV, TGF-β is the strongest inducer of myofibroblast formation. Indeed, in response to TGF-β, quiescent VICs (qVICs) differentiate into activated VICs (aVICs) exhibiting a myofibroblastic phenotype characterized by the expression of α-smooth muscle actin (*α*-SMA). These aVICs proliferate and secrete matrix metalloproteinases (MMPs), driving ECM remodeling, leaflet thickening, and increased stiffness (18, 22, 24). In the early stages of CAVD, TGF-β also promotes EndMT, leading to the formation of myofibroblasts from VECs (15, 25). During this process, VECs downregulate their expression of endothelial markers such as CD31 and VE-cadherin, while upregulating mesenchymal markers like α-SMA. EndMT can also be induced by inflammatory cytokines (e.g., IFNγ, IL-6, TNF-α, or LPS) (25), disturbed flow patterns (26), and metabolic stressors such as oxidized LDL or hyperglycemia (27). The presence of neovessels and inflammatory infiltrates in sclerotic valves supports the view that leaflet thickening results at least in part from an active and chronic inflammatory process (28–30).

### 3.3 Main mechanisms driving calcification

Over time, aVICs may gradually downregulate α-SMA and transition into osteoblast-like VICs (obVICs), acquiring the capacity to deposit a bone-like, calcifiable matrix. This calcification process is tightly regulated by the balance between inhibitors that prevent calcium phosphate (Ca/P) deposition and activators that drive VIC osteogenic differentiation. Key inhibitors include pyrophosphate (PPi), which directly interferes with hydroxyapatite formation, and matrix Gla protein (MGP) together with Fetuin-A, which stabilize calcium and phosphate ions into amorphous calciprotein particles (CPPs) to promote Ca/P clearance and prevent ectopic calcification (31–36). Activators include inflammation, oxidative stress, and local elevations in calcium and phosphorus, all of which drive VICs transition toward obVICs expressing osteogenic markers such as Bone Morphogenetic Protein 2 (BMP2), Runt-related transcription factor 2 (RUNX2), and alkaline phosphatase (ALP). BMP2 induces RUNX2, a master transcription factor regulating osteogenic genes including ALP, osteopontin (OPN), type I collagen, and osteocalcin (OCN). ALP activity promotes mineral deposition by hydrolyzing PPi into inorganic phosphate (37). Accordingly, calcifying VICs exhibit high ALP activity and reduced PPi levels (38).

Osteoblast-like VICs release extracellular vesicles (EVs) enriched in pro-calcific ectonucleotidases such as ALP, ENPP1 (ectonucleotide pyrophosphatase/phosphodiesterase 1), and 5′-nucleotidase (37, 39–42). ENPP1 and 5′-nucleotidase hydrolyze ATP to produce PPi, which is subsequently degraded by ALP (40, 41). Because ATP also acts as a survival signal via P2Y2 receptors, its degradation by ENPP1 may favor VIC apoptosis (40). Apoptotic bodies, which resemble calcifying EVs, further contribute to matrix mineralization. In collagen-rich areas, EVs cluster to form macrocalcifications, whereas in collagen-poor areas, EVs remain dispersed, leading to microcalcifications (43). Annexin A1, a calcium-binding protein secreted by obVICs, facilitates EVs aggregation and calcification (44).

Early calcific nodules often co-localize with lipid-rich regions and consist of hydroxyapatite embedded within a matrix enriched in collagen, OPN, and other bone matrix proteins (20, 45). In advanced stages of CAVD, valve tissue may display cartilage- and bone-like features, including lamellar bone, hematopoietic marrow elements, neovascularization, and even microfractures (20, 46). These ossific changes markedly reduce leaflet compliance and accelerate stenotic progression.

### 3.4 Side-specific AV remodeling

Aortic valve remodelling predominantly affects the aortic side of the leaflets, where cells and the ECM are exposed to complex and disturbed hemodynamic forces (47). On this side, shear stress induces the expression of endothelial adhesion molecules such as VCAM-1 and ICAM-1 via TGF-β1– and BMP-4–dependent pathways (48), thereby promoting monocyte adhesion, rolling, and infiltration. By contrast, these inflammatory responses are absent on the ventricular surface, despite exposure to shear forces. This side-specific inflammation is thought to arise from differences in local flow dynamics, with relatively stable flow on the ventricular side vs. disturbed flow on the aortic side. Histologically, calcification occurs more frequently at the base and center of the valve than within the leaflet cusps (49), consistent with these regions experiencing greater mechanical stress, which contributes to age-related calcification (1, 50). Notably, in tricuspid CAVD, macrophages infiltration mainly occurs in the valve base and center, surrounding calcified regions (49), reinforcing the idea that macrophages may be key modulators of the calcification process.

## 4 From monocyte infiltration to macrophage differentiation

**Figure 1:**
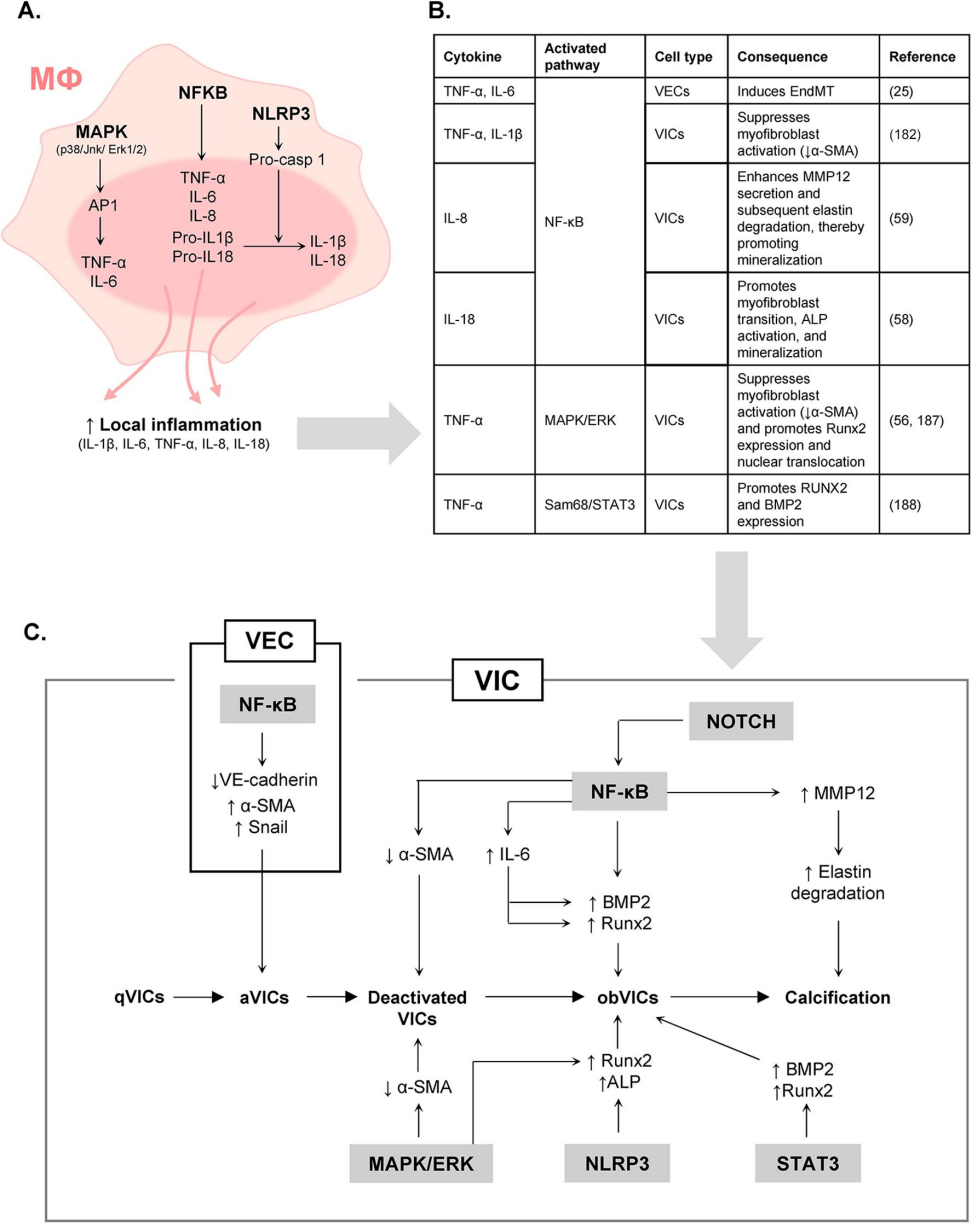
Inflammatory signaling in CAVD: role of pro-inflammatory cytokines. **(A)** Main signaling pathways involved in cytokine secretion by pro-inflammatory macrophages. **(B)** Key signaling pathways through which pro-inflammatory cytokines influence the phenotype of valvular cells. **(C)** Identification of the main inflammation-associated signaling pathways involved in the phenotypic transitions of VICs and VECs. Activation of NF-κB signaling (in particular by TNF-α and IL-1β) can suppress myofibroblast activation in VICs (182). NF-κB activation has also been reported to induce BMP2 (183) and RUNX2 (184) expression, thereby promoting VIC mineralization. In VICs, NF-κB signaling has further been associated with osteogenic differentiation, a phenomenon that seems linked to increased secretion of IL6 (128, 185). In addition, NF-κB activation has been linked to MMP12-mediated elastin degradation in response to IL-8, thus contributing to calcification (59). Moreover, Notch signaling has been shown to amplify NF-κB–induced BMP2 production in VICs (183). In VECs, activation of NF-κB signaling by TNF-α or IL-6 promotes EndMT, characterized by decreased VE-cadherin expression together with increased α-SMA and Snail expression (25). Beyond NF-κB, NLRP3 activation in VICs was recently demonstrated to promote RUNX2 and ALP expression, as well as the upregulation of inflammatory markers such as ICAM1 and VCAM1 (186). In VICs, activation of the MAPK/ERK pathway by TNF-α favors deactivation and promotes osteogenic differentiation (56, 187). Finally, TNF-α–mediated activation of the Sam68 pathway has been shown to promote the osteogenic differentiation of aortic VICs (188).

Once infiltrated into the leaflet, monocytes rapidly differentiate into macrophages that adopt distinct functional phenotypes depending on local cues. Th1 cytokines, such as interferon-gamma (IFN-γ), drive them toward a pro-inflammatory profile characterized by the release of IL-1β, IL-6, TNF-α, IL-8 and reactive oxygen species. Three major signaling pathways are primarily involved in macrophages production of pro-inflammatory cytokines. Activation of the MAPK pathway promotes the production of TNF-α and IL-6. Activation of the NF-κB pathway induces the expression of TNF-α, IL-6, IL-8, and the precursors pro-IL-1β and pro-IL-18. Subsequent activation of the NLRP3–pro-caspase-1 axis enables the cleavage of these precursors, resulting in the secretion of mature IL-1β and IL-18 (Figure 1A). Pro-inflammatory macrophages are typically identified by the expression of CD11c, CD80, CD86, CD64, CD16 and CD32, with inducible nitric oxide synthase (iNOS) also serving as a marker. In contrast, Th2 cytokines (e.g., IL-4 and IL-13) drive macrophages toward an immunomodulatory phenotype, marked by the secretion of IL-1 receptor antagonist (IL-1ra), IL-10, CCL22, TGF-*β*1 and alternative macrophage activation-associated CC chemokine-1 (AMAC-1). CD163 and CD206 are key markers of immunomodulatory macrophages in humans. In mice, Ym1, Arg1, and Fizz1 are among the most widely used. Importantly, macrophages polarization is highly reversible: *in vitro*, cells can switch from one phenotype to another within 24 h when exposed to appropriate cytokines (51).

Macrophage infiltration is increased in calcified compared to non-calcified AVs (52). Interestingly, both pro-inflammatory (TNF-α, IL-12α, and IL-6) and immunomodulatory (TGF-β1, IL-10, and AMAC-1) cytokines are upregulated in CAVD samples. Nevertheless, the pro-inflammatory subset remains the predominant macrophage population in CAVD (52, 53), prompting researchers to suggest that this shift may be detrimental to valve health.

## 5 Impact of pro-inflammatory macrophages on AV remodelling

### 5.1 Role of inflammatory cytokines

Pro-inflammatory cytokines are key drivers of ECM degradation, remodeling, and calcification (Figure 1B). TNF-α and IL-6 have been shown to upregulate BMP2 and RUNX2 in VICs, promoting their mineralization (30, 54–56). IL-1β stimulates VIC proliferation and the secretion of MMP1 and MMP2 (57). IL-18 induces NF-κB expression, myofibroblast transition, ALP activation, and mineralization in VICs (58). IL-8 activates NF-κB in VICs, increasing MMP12 secretion and elastin degradation, thereby promoting VIC mineralization without affecting their osteogenic transition (59).

As a consequence, conditioned medium (CM) from pro-inflammatory macrophages enhances VIC expression of several osteoblastic markers, including BMP2, ALP, and OPN, as well as their mineralization, compared to CM from unpolarized macrophages (52). These effects are blocked by neutralizing antibodies against TNF-α or IL-6, indicating that the pro-osteogenic action of pro-inflammatory macrophages on VICs is mediated, at least in part, by TNF-α and IL-6. Interestingly, TNF-α and IL-1β in pro-inflammatory macrophage CM can deactivate aVICs, reducing αSMA expression while simultaneously promoting proliferation (60). Notably, female VICs retain higher *α*SMA levels than male VICs following exposure to CM from pro-inflammatory macrophages (56), suggesting a sex-specific sensitivity to macrophages activity. In 2020, Grim et al. demonstrated that IL-6 in macrophages-CM—but not TNF-α or IL-1β—promotes aVICs differenciation toward obVICs, marked by increased nuclear translocation of RUNX2 and elevated OPN expression (60). More recently, Vélez et al. showed that TNF-α can also induce RUNX2 nuclear translocation, but only in primary VIC from women (56). These findings indicate that pro-inflammatory macrophages may contribute to the transition from fibrosis to calcification in AS and highlight the importance of considering sex as a biological variable in AS research. Interestingly, CM from VICs isolated from CAVD samples reduced MMP-9 activity and increased type I collagen production in pro-inflammatory macrophages compared to CM from healthy AVs, suggesting a bidirectional crosstalk in which macrophages promote the transition of qVICs into aVICs, which in turn enhance the pro-fibrotic properties of macrophages (61).

*In vitro*, IL-6 and TNF-α induce EndMT of primary VECs and promote cell invasion in a dose-dependent manner via an Akt/NF-κB–dependent pathway (25). Accordingly, it is conceivable that local macrophage-driven elevations of TNF-α and IL-6 may contribute to fibrosis and calcification by promoting EndMT. Investigating the effects of CM from pro-inflammatory macrophages on EndMT could help validate this hypothesis. Furthermore, Xian et al. demonstrated that M1 macrophages markedly increased endothelial expression of VLA4/VCAM-1 (adhesion molecules mediating leukocyte–endothelium interactions) and RAC1/p-PYK2/p-VE-cadherin (signaling molecules regulating cytoskeletal dynamics, kinase activity, and endothelial permeability), accompanied by a significant increase in endothelial permeability (62). Confirming these findings, they observed a substantial presence of M1 macrophages in AVs from rats with rheumatic heart disease, associated with elevated VLA4/VCAM-1 and RAC1/p-PYK2/p-VE-cadherin expression, suggesting that M1 macrophages may amplify immune cell infiltration by compromising VEC integrity. However, as in many other studies, the endothelial cells used in this study were derived from human umbilical veins rather than from valvular endothelium. Importantly, recent transcriptomic (63) and functional (64) analyses show that vascular endothelial cells differ substantially from VECs, indicating that results obtained with generic vascular endothelial cells may not be directly translatable. Future studies should prioritize the use of primary VECs to accurately investigate their crosstalk with macrophages. The main signaling pathways associated with pro-inflammatory remodeling are shown in Figures 1B,C.

### 5.2 Intrinsic osteogenic activity of pro-inflammatory macrophages

Pro-inflammatory macrophages exhibit constitutive activation of BMP-2-dependent signalling pathways (65), suggesting the existence of an auto/paracrine mechanism in which BMP-2 secreted by pro-inflammatory macrophages sustains constitutive BMP-2 signalling in neighbouring cells. Supporting this hypothesis, the use of an antibody targeting BMP2 prevented the induction of ALP expression in human mesenchymal stem cells by CM from pro-inflammatory macrophages (66). Cellular Communication Network Factor 3 (CCN3) appears to regulate macrophage BMP2 production, since bone marrow-derived macrophages (BMDMs) from CCN3-deficient mice exhibit increased BMP2 synthesis and secretion (67). CM from these macrophages promoted VIC osteogenic transition and mineralization, an effect blocked by BMP2 neutralization. Consistently, mice with myeloid-specific CCN3 deficiency display exacerbated valvular calcification and dysfunction (67). Together, these findings identify CCN3 as a potential anti-calcific factor through its regulatory role in macrophage osteogenic activity and suggest that targeting BMP2 may represent a promising strategy to prevent macrophage-induced valvular calcification.

### 5.3 Role of macrophages-derived EVs

In 2022, Xia et al. demonstrated that pro-inflammatory macrophages communicate with VICs via EVs secretion (68). Internalization of these EVs significantly increased calcium nodule formation and the expression of osteogenic genes in VICs, including RUNX2, BMP2, OPN, compared to EVs from control macrophages. VICs exposed to EVs from pro-inflammatory macrophages also showed higher *α*-SMA and collagen I levels, indicating that these EVs promote both osteogenic and fibrotic processes. Xia et al. further showed that these EVs deliver tRNA-derived small RNAs (tsRNAs)—a novel class of noncoding RNAs implicated in cardiovascular diseases (69, 70). Among them, tsRNA-5006c, which regulates autophagy, was markedly enriched in EVs from pro-inflammatory macrophages. Deletion of tsRNA-5006c reduced calcium nodule formation and lowered the expression of osteogenic (RUNX2, BMP2, OPN) and fibrotic (collagen I, *α*-SMA) markers in VICs, highlighting its key role in driving VIC phenotypic differentiation. Notably, VICs exposed to these EVs showed enhanced mitophagy, which disappeared upon tsRNA-5006c inhibition. These findings are consistent with previous studies linking excessive mitophagy and autophagy to osteogenic differentiation and CAVD progression (71).

In CAVD, pro-inflammatory polarization is accompanied by the upregulation of miR-214, a microRNA essential for pro-inflammatory polarization (72). Increased miR-214 levels in AV samples correlate with decreased expression of its target gene TWIST-1, a transcription factor that inhibits osteoblastic transdifferentiation of human VICs by antagonizing RUNX2 (73). This has led to the hypothesis that macrophage-derived miR-214 promotes CAVD. Supporting this, co-culture with pro-inflammatory macrophages or their EVs reduced TWIST-1 expression in VICs, accompanied by increased ALP activity and elevated OCN expression. Conversely, VICs exposed to EVs from miR-214-silenced pro-inflammatory macrophages showed higher TWIST-1 expression and reduced ALP activity and OCN expression compared to those treated with wild-type pro-inflammatory EVs. These effects were abolished when VICs were pre-treated with TWIST-1 siRNA. Intravenous injection of a miR-214 inhibitor in hypercholesterolemic apoE-/- mice increased valvular TWIST-1 expression and markedly reduced AV calcification. Together, these results suggest that pro-inflammatory macrophages deliver miR-214 via EVs, thereby downregulating TWIST-1 and promoting VIC mineralization. This discovery is particularly relevant given emerging therapeutic strategies involving engineered EVs (74).

### 5.4 Impact of physical contacts between pro-inflammatory macrophages and VICs

**Figure 2:**
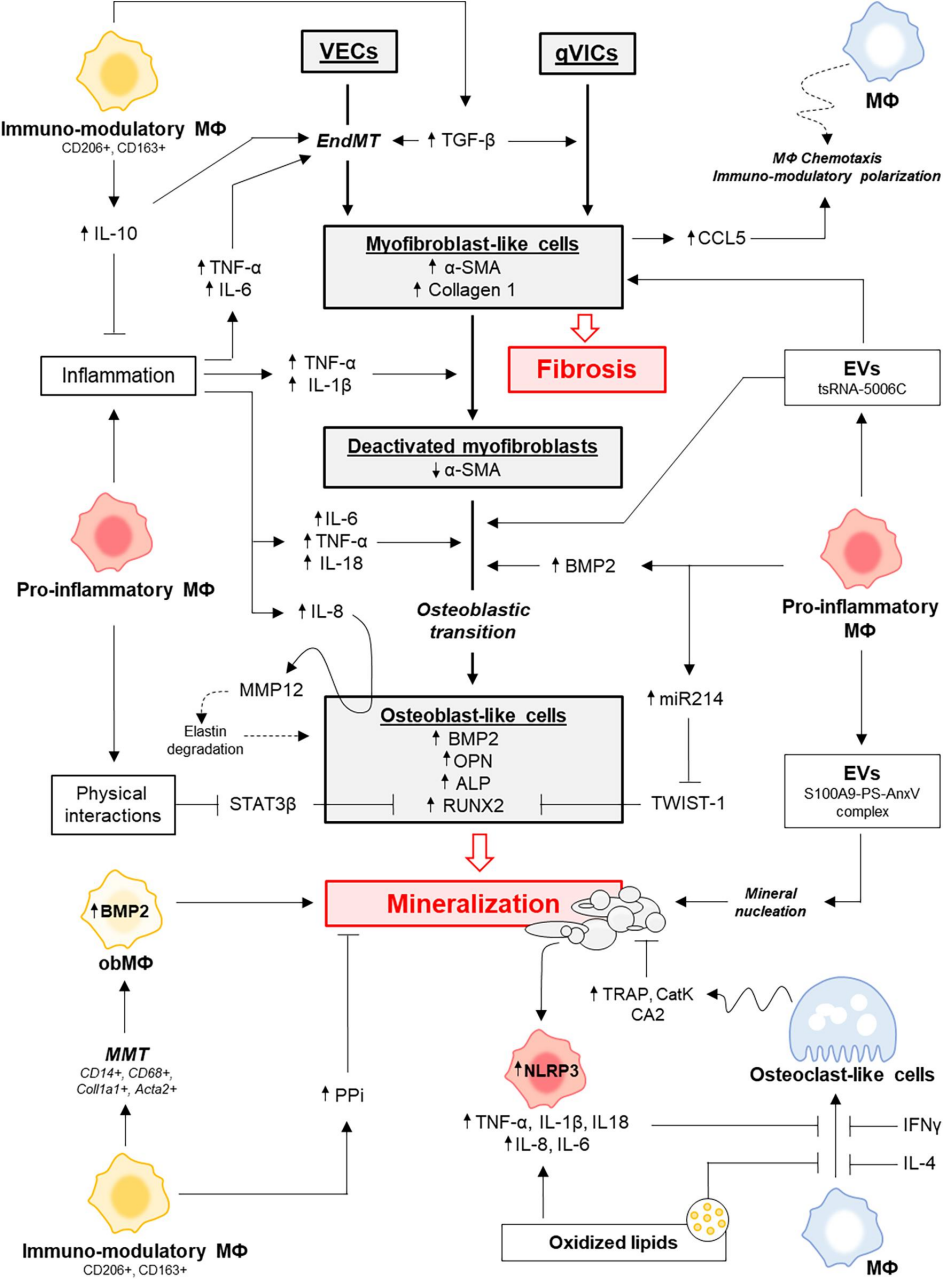
Overview of the main mechanisms by which macrophage subtypes influence the fibrocalcific remodeling of aortic valve leaflets. ALP, alkaline phosphatase; α-SMA, α-smooth muscle actin; BMP2, bone morphogenetic protein 2; CA2, carbonic anhydrase II; CatK, cathespin K; CCL5, C-C Motif Chemokine Ligand 5; EVs, extracellular vesicles; IFNγ, interferon γ; IL-4, interleukin 4; IL-1β, interleukin 1β; IL-6, interleukin 6; IL-10, interleukin 10; MФ, macrophages; MMP12, matrix metalloproteinase 12; NLRP3, NOD-Like Receptor Pyrin Domain-Containing Protein 3; obMΦ, macrophages with an osteoblast-like activity; OPN, osteopontin; PPi, pyrophosphate; qVICs, quiescent VICs; RUNX2, Runt-related transcription factor 2; TGF-β, transforming growth factor β; TNF-α, tumor necrosis factor α; TRAP, Tartrate-resistant acid phosphatase.

Physical interactions between macrophages and VICs enhance the calcification process. Indeed, direct co-culture with macrophages further promotes the osteogenic transition of VICs, as evidenced by increased RUNX2 expression, compared to indirect co-culture in transwell systems without physical contact (75). This physical interaction is associated with a marked decrease in VIC expression of STAT3β, an alternatively spliced isoform of STAT3 that binds and inhibits RUNX2 (75, 76). Consistent with these findings, STAT3β expression negatively correlates with RUNX2 levels in calcified regions of human AVs (75), suggesting that reduced STAT3β expression relieves the inhibitory effect on RUNX2, thereby promoting VIC osteogenic transition and mineralization. The main pathways through which pro-inflammatory macrophages drive fibrocalcific remodeling of the AV are summarized in Figure 2.

## 6 Impact of immunomodulatory macrophages on AV remodelling

### 6.1 Evidence linking immunomodulatory cytokines to AV remodelling

Immunomodulatory macrophages contribute to the resolution of inflammation and tissue repair primarily through the secretion of anti-inflammatory cytokines such as IL-10 and TGF-β (77, 78), which has long suggested a protective role against CAVD. Indeed, IL-10 suppresses Th1 responses and inhibits monocyte functions, including the production of pro-inflammatory cytokines such as TNF-α and IL-8 (79). In CAVD patients, plasma IL-10 levels inversely correlate with valve inflammation and degeneration, and IL-10 gene polymorphisms have been linked to CAVD (80). TGF-β, a key regulator of fibrosis and immune homeostasis, is elevated in calcified compared to noncalcified control cusps (81, 82). *In vitro*, recombinant TGF-β promotes the differentiation of qVICs into aVICs and enhances VICs mobility, aggregation, as well as the formation of nodules enriched in ALP, leading to mineralization (83–85). In this model, blockade of TGF-β1-induced apoptosis with a caspase inhibitor significantly reduced mineralization but did not affect nodule formation, whereas cytochalasin D, an actin-depolymerizing agent, inhibited nodule formation without impacting calcification. Together these findings suggest that targeting TGF-β activity could protect the valve from fibro-calcic remodelling. The ability of IL-10 and TGF-β to promote EndMT and neoangiogenesis reinforces the idea that immunomodulatory macrophages can detrimentally contribute to valvular disease (82, 86).

In 2023, Wu et al. showed that CM from immunomodulatory macrophages induces VIC expression of *α*-SMA without affecting RUNX2 levels (87). This indicates that secretions from immunomodulatory macrophages may drive myofibroblast activation but do not directly trigger osteogenic differentiation in VICs. Although histological analyses of CAVD samples confirm colocalization of CD163 and *α*-SMA (87), no mechanistic study to date has demonstrated that factors secreted by immunomodulatory macrophages, particularly TGF-β, directly drive myofibroblastic differentiation and fibrosis. Further research is needed to clarify this issue.

Interestingly, CM from aVICs promotes macrophage chemotaxis by increasing CCL5 secretion via the AKT pathway (87). Macrophage adhesion to aVICs decreases upon blocking CD44, the main receptor for hyaluronic acid (HA), indicating that aVICs not only recruit immune cells but also stabilize cell contacts through the HA/CD44 complex. Consistently, both HA and CD44 are upregulated in fibrotic tissues (88). Notably, CM from aVICs drives macrophage polarization toward an immunomodulatory phenotype, marked by increased expression of CD206, CD163, TGF-β, and IL-10 and decreased IL-1β expression; this effect is prevented by inhibiting hyaluronan synthase-2. Together, these findings reveal a positive feedback loop in which aVICs promote the recruitment and polarization of macrophages toward an immunomodulatory phenotype, which in turn further stimulates aVIC activation and drives CAVD progression. Targeting aVIC–macrophage interactions, particularly via the HA/CD44 axis, may therefore represent a promising therapeutic strategy.

In calcified regions of CAVD samples, the immunomodulatory markers CD163 and CD206 strongly correlate with TLR7 mRNA levels (89). *Ex vivo* stimulation of AV tissue with the TLR7 ligand imiquimod induced the secretion of IL-10, TNF-α, and GM-CSF. *In vitro* experiments suggest that macrophages are the main source of these cytokines. Together, these findings link TLR7 activation in CAVD to immunomodulatory and anti-inflammatory responses. Further studies are needed to clarify the relationship between TLR7 signaling and immunomodulatory macrophage pathways in human AVs.

### 6.2 Secretion of pyrophosphate

Macrophages produce PPi through ATP hydrolysis mediated by eNPP1. In 2016, Villa-Bellosta et al. reported that immunomodulatory macrophages promote PPi synthesis more efficiently than pro-inflammatory macrophages by enhancing ATP release and upregulating eNPP1 (51). While this mechanism protected VSMCs from calcification *in vitro*, its relevance to VICs remains unclear. Since PPi inhibits calcification through a physicochemical rather than a cellular mechanism, a similar protective role in the AV is plausible but requires further investigation.

### 6.3 Macrophage-to-mesenchymal transition (MMT)

Although immunomodulatory macrophages do not seem to directly promote the transition of aVICs into obVICs *in vitro*, emerging evidence suggests that they may themselves contribute to the pool of osteoblast-like cells through a process known as macrophage-to-mesenchymal transition (MMT) (90). In 2023, Lyu et al. used single cell RNA sequencing (scRNA-seq) on calcified bicuspid AVs and identified a subpopulation co-expressing monocytic markers (e.g., CD14, CD68) and mesenchymal markers (e.g., COL1A1, ACTA2), with activation of ECM organization and calcification-related pathways (90). Termed “macrophage-derived stromal cells”, these cells were shown to originate from CD206^+^ immunomodulatory-like macrophages undergoing MMT. During this process, macrophages upregulated myofibroblastic and osteogenic markers—including BMP2, ASPN, and POSTN—and progressively differentiated into osteoblast-like cells capable of mineralization. These findings align with the 2020 study by Oba et al., who reported increased CD163+ and CD206+ macrophages in calcified (*n* = 36) compared to non-calcified (*n* = 6) AVs, with most CD206+ macrophages expressing BMP2 (49). Figure 2 summarizes the main pathways through which immunomodulatory macrophages influence fibrocalcific remodeling of the AV.

## 7 Role of M(Hb) macrophages in AV remodeling

While this review primarily emphasizes the roles of pro-inflammatory and immunomodulatory macrophages in CAVD, numerous other subsets exist whose contributions to AS remain largely unexplored. Among them, M(Hb) macrophages—an anti-inflammatory population resistant to cholesterol accumulation—may play a significant role in AV remodelling. These cells typically emerge in vascular tissue following plaque hemorrhage, in response to hemoglobin (Hb)/haptoglobin (Hp) complexes, and are characterized by high expression of heme oxygenase-1 (HO-1). In 2023, Sakamoto et al. demonstrated that M(Hb) macrophages inhibit VSMC calcification compared with unpolarized macrophages (91), through the secretion of hyaluronan, a glycosaminoglycan that prevents Ca/P deposition. Inhibition of hyaluronan synthesis abolished this protective effect. Notably, HO-1+ macrophages are enriched in calcified compared with non-calcified AV tissue (49). Whether these cells arise as a compensatory mechanism to protect VICs from calcification, as observed in VSMCs, remains unknown. Intriguingly, some HO-1+ macrophages in AVs also express BMP2 (49), suggesting that this subset may not only exert protective functions but also contribute to osteogenic activity. Further studies are therefore needed to clarify the precise role of M(Hb) macrophages and other underexplored subsets in AS pathogenesis and progression.

## 8 Crosstalk between macrophages and calcification crystals

*In vitro*, exposure of naïve macrophages to Ca/P nanocrystals induces polarization toward a pro-inflammatory phenotype characterized by increased iNOS expression and secretion of cytokines such as TNF-α, which further amplify calcification (92, 93). Ca/P nanocrystals also activate the NLR family pyrin domain containing 3 (NLRP3) inflammasome in THP-1 cells and murine BMDMs, leading to IL-1β release (93). Human monocyte-derived macrophages can engulf Ca/P microcrystals within intracellular vacuoles and respond by producing pro-inflammatory mediators including TNF-α, IL-1β, and IL-8, which in turn activate endothelial cells and enhance leukocyte adhesion under flow conditions (94). Interestingly, the pro-inflammatory potential of Ca/P nanocrystals inversely correlates with their size: crystals measuring 1–2 µm in diameter with pore sizes of 10–50 Å elicit the strongest TNF-α response (95), suggesting that small, early-stage nanocrystals are more inflammatory than larger bone-like deposits. These findings support a model in which calcification and inflammation reinforce each other in a deleterious feedback loop, driving CAVD progression.

MGP and fetuin-A stabilize nascent Ca/P clusters by forming CPPs, which facilitate Ca/P clearance and prevent ectopic calcification. *In vitro*, CPPs induce lower cytokine secretion and cause less macrophage apoptosis than hydroxyapatite crystals of similar size and calcium content (32). Clinically, CAVD progression is associated with reduced serum fetuin-A levels (96), likely decreasing CPP formation and promoting accumulation of large, pro-inflammatory hydroxyapatite deposits. Interestingly, fetuin-A is elevated in CAVD samples compared to healthy AVs, suggesting a compensatory mechanism whereby increased calcification drives fetuin-A accumulation in the AV to limit further mineral deposition (97).

Macrophages release EVs ranging from 30 to 300 nm. Upon Ca/P stimulation, these EVs show increased ALP activity and promote hydroxyapatite nucleation. They carry typical exosomal markers such as CD9 and TSG101, along with S100A9, phosphatidylserine (PS), and annexin V (Anx5), resembling chondrocyte-derived EVs involved in bone mineralization (98, 99). Ca/P exposure enhances the interaction between S100A9 and Anx5 and promotes PS externalization on EVs membrane, suggesting that mineral nucleation occurs at the EV surface. Silencing S100A9 or using S100A9-deficient mice reduces EV-mediated mineralization, whereas S100A9 stimulation increases calcification, highlighting the key role of the PS–Anx5–S100A9 complex in HA formation (100). Ca/P-stimulated macrophages upregulate pro-inflammatory markers (iNOS, IL-6) and downregulate immunomodulatory markers (CD206, ARG1), indicating that pro-calcific EVs mainly originate from pro-inflammatory macrophages. Studies have shown that High mobility group box 1 (HMGB1), a nuclear protein released by stressed cells and enriched in regions of macrophage infiltration and calcification in AS, promotes EVs secretion. HMGB1 activates the receptor for advanced glycation end-products (RAGE), triggering p38 MAPK/nSMase2 signaling in macrophages, which enhances EVs release and contributes to mineralization *in vitro* and *in vivo* (101–104). Collectively, these data highlight a dynamic, reciprocal interaction between macrophages and Ca/P crystals in CAVD progression (Figure 2). Understanding the molecular mechanisms governing this crosstalk could reveal novel therapeutic targets to halt AV calcification.

## 9 Macrophages as precursors of osteoclast-like cells

About 13% of calcified AVs contain true bone with osteoblasts and osteoclasts (105). Macrophages are considered precursors of these osteoclast-like cells, as both originate from the same hematopoietic lineage. Upon stimulation with macrophage colony-stimulating factor (M-CSF) and receptor activator of nuclear factor κB ligand (RANKL), macrophages can differentiate into multinucleated osteoclasts specialized in bone resorption. Both RANKL and MCSF are present in human stenotic AVs (106, 107), suggesting that an osteoclastogenesis-like process may occur locally. Supporting this, tartrate-resistant acid phosphatase (TRAP) and cathepsin K—enzymes associated with bone resorption—are detected in calcified but not in non-calcified AVs (107), and osteoclasts-like cells can actively reduce mineral deposits on pre-calcified aortic elastin *in vitro* (108, 109). In addition, macrophages express high levels of carbonic anhydrase 2 (CA2) (110), an enzyme critical for mineral dissolution by osteoclasts, as it acidifies the resorption lacuna by generating protons. In cell-free assays, macrophages decalcification capacity correlates with CA2 expression (110). However, AV calcification does not appear to regress *in vivo*, suggesting that the valvular microenvironment impairs osteoclast-like activity. Supporting this, IL-18, whose expression is upregulated in CAVD and correlates with disease severity (111), inhibits osteoclastogenesis *in vitro* (112). Similarly, oxidized LDL (oxLDL) suppresses RANKL-induced TRAP activity and resorptive activity in human peripheral blood mononuclear cells (PBMCs) and mouse RAW 264.7 macrophages (113) (Figure 2).

Recent studies have investigated whether osteoclast-like cells arise preferentially from pro-inflammatory macrophages or immunomodulatory macrophage. In 2017, Chinetti-Gbaguidi et al. showed that IL-4-induced polarization toward an immunomodulatory phenotype reduces the ability of macrophages to form TRAP-positive osteoclast-like cells. These cells exhibit low cathepsin K expression, diminished TRAP activity, and defective resorptive capacity. Mechanistically, IL-4 downregulates NFATc1, the transcriptional regulator of cathepsin K, through enrichment of the repressive histone mark H3K27me3 at its promoter (114).

Conversely, Nagy et al. demonstrated that pro-inflammatory signals can also impair osteoclast-like differentiation (107). They reported that the exposure of calcified AV tissues to a T-cell activation cocktail increased IFN-γ production and reduced RANKL, TRAP, and cathepsin K expression. PBMCs cultured with CM from these tissues formed fewer osteoclast-like cells with reduced resorptive capacity, an effect that was reversed by anti-IFN-*γ* antibodies. Barinda et al. further showed that pro-inflammatory polarization or treatment with vasoactive peptides (endothelin-1, angiotensin II) or cytokines (TNF-α, IL-1β) downregulate CA2 expression in macrophages (110). Similarly, Yamaguchi et al. reported that co-culture with pro-inflammatory macrophages suppresses RANKL-induced osteoclastogenesis, through soluble factors including IFN-γ and IL-12, which inhibit NFATc1 expression and promote osteoclast precursor apoptosis (115).

Collectively, these findings indicate that both immunomodulatory and pro-inflammatory signals can hinder the differentiation of macrophages into functional osteoclast-like cells. Therefore, the origins and developmental pathways of these resorptive cells in CAVD remains incompletely understood. Large-scale approaches such as scRNA-seq may help clarify their identity and trajectory. Strategies aimed at enhancing macrophage osteoclastic capacity through targeted pharmacological interventions may offer a novel approach to counteract valvular calcification, although the impact on systemic bone physiology requires careful evaluation.

## 10 Modulating macrophage activity to slow aortic stenosis

Recent advances in understanding how macrophages influence AV fibrosis and calcification have identified novel molecular targets and therapeutic strategies. These insights have spurred both the development of new pharmacologic approaches and renewed interest in repurposing existing drugs for potential use in CAVD. Table 1 summarizes emerging molecular targets and therapeutic strategies aimed at modulating macrophage function to prevent AV remodeling.

**Table 1:** Emerging molecular targets and therapeutic strategies modulating macrophage activity in CAVD. .

Targets	Mechanisms of action	References	Potential therapy	Evidence of efficiency in AS			
				Preclinical	References	Clinical	References
TNF-α	Secretion of TNF-α by pro-inflammatory macrophages activates EndMT, and promotes myofibroblasts deactivation as well as ostegenic differenciation	(25, 30, 52, 54, 56, 60, 189)	Antibodies against TNFα (infliximab, adalimumab, golimumab, certolizumab pegol …)	*In vitro*, antibodies neutralizing TNF-α prevent the osteogenic transition and mineralization of VICs induced by CM from pro-inflammatory macrophages	(52)	No clinical data available	
IL-6	Secretion of IL-6 by pro-inflammatory macrophages activates EndMT and promotes VICs ostegenic differenciation	(25, 52, 60, 126)	Antibodies against IL6 (ziltivekimab, clazakizumab, …)	*In vitro*, neutralizing antibodies against IL-6 prevent the osteogenic transition and mineralization of VICs induced either by CM from pro-inflammatory macrophages or by the uremic toxin indoxyl sulfate.	(52, 128)	Ziltivekimab and Clazakizumab are currently being evaluated for their cardiovascular effects in patients with CKD and persistent inflammation.	(130–132)
						No clinical data available regarding AS	
			Antibody angainst IL-6 receptor (Tocilizumab)	No preclinical data available		Mendelian randomization studies have associated genetically predicted tocilizumab treatment with a reduced risk of AS	(129)
IL-1β	Secretion of IL-1β by pro-inflammatory macrophages promotes myofibroblasts proliferation, secretion of MMP1 and 2 as well as myofibroblasts deactivation	(57, 60)	Recombinant IL-1 receptor antagonist (Anakinra)	Deficiency of interleukin-1 receptor antagonist induces AV disease in BALB/c mice	(190)	Has shown benefits for cardiorespiratory function and quality of life in HF, particularly among patients with preserved ejection fraction.	(136, 137)
						No clinical data available regarding AS	
			Fusion protein targeting IL-1 receptors (rilonacept)	No preclinical data available		No clinical data available	
			Monoclonal antibody against IL-1β (Canakinumab)	No preclinical data available		Reduced systemic inflammation and improved HF outcomes in post-myocardial infarction patients.	(135)
						No clinical data available regarding AS	
IL-18	Secretion of IL-18 by pro-inflammatory macrophages induces NF-*κ*B expression, myofibroblast transition, ALP activation, and VIC calcification	(58)	Recombinant IL-18 binding proteins, anti-IL18 antibodies, IL-18 receptor antagonists	No preclinical data available		No clinical data available	
IL-8	Secretion of IL-8 by pro-inflammatory macrophages, and its subsequent binding to CXCR2, promotes MMP-12 secretion, contributing to elastin degradation and, consequently, to VIC calcification. Moreover, IL-8 promotes the pro-inflammatory polarization of macrophages.	(59, 191)	Antagonist of CXCR2 (Navarixin)	Prevents VICs mineralization *in vitro*, and prevents AV calcification in a rat model of CKD.	(59)	No clinical data available	
			Antibody angainst IL-8 (ividalimab)	No preclinical data available		No clinical data available	
NLRP3	Activation of the NLRP3 pathway in macrophages mediates caspase-1 activation and the secretion of IL-1β and IL-18	(192)	CY-09	Administration of CY-09 in ApoE-/- mice fed a high-fat diet prevented pro-inflammatory polarization of macrophages in the AV, inhibited osteogenic transition and AV mineralization, and improved AV function.	(140)	No clinical data available	
			Non-specific NLRP3 inhibitor (Colchicine)	No preclinical data available		Colchicine reduces ischemic events following myocardial infarction	(144)
						Colchicine has not demonstrated efficacy in improving outcomes in HF	(193)
						No clinical data available regarding AS	
			Promoting TREM2 activity	TREM2 suppression triggers NLRP3 inflammasome activation, resulting in increased secretion of IL-1β, TNF-α, IL-6 and ROS. Inhibition of TREM2 via adeno-associated virus serotype 9 (AAV9) vectors delivering shRNA in ApoE-/- mice fed a high-fat diet exacerbated AV calcification.	(142)	No clinical data available regarding AS	
				AL002a—a novel agonistic antibody targeting TREM2 that has demonstrated efficacy in enhancing plaque stability in murine models of atherosclerosis	(143)		
IL-37	Prevents pro-inflammatory polarization and promotes immunomodulatory polarization of macrophages by inhibiting NF-κB and Notch1 signaling	(120)	Recombinant IL-37	Recombinant IL-37 dampens pro-inflammatory polarization and favors immunomodulatory polarization of macrophages	(120)	No clinical data available	
				IL-37 suppresses the osteogenic responses of human VICs *in vitro* and alleviates valve lesions in mice	(121)		
				Recombinant IL-37 also exerts an anti-inflammatory effect on human VICs *in vitro*, thereby preventing their mineralization	(122, 123)		
PPi	Secretion of PPi by immunomodulatory macrophages prevents Ca/P deposition	(51)	PPi analogs (Bisphosphonates ibandronate, alendronate, zoledronate, and etidronate)	Zoledronic acid halted the progression of AV calcification induced by excessive vitamin D intake in a New Zealand rabbit model	(148)	Bisphosphonate use was independently linked to slower progression of mild AS in individuals with preserved kidney function	(149)
						Alendronate (70 mg weekly over 24 months) slowed stenosis progression and improved clinical prognosis in patients with AS and concurrent osteoporosis	(150)
						Alendronic acid (70 mg weekly for 24 months) had no significant effect on AV calcification progression in patients with established calcific AS	(151)
RANKL	Promotes pro-inflammatory polarization of macrophages, and subsequent secretion of IL-6 and TNF-α. Promotes VICs osteogenic transition and minéralization	(106, 154)	Monoclonal antibody targeting RANKL (Denosumab)	Denosumab prevents VICs mineralization *in vitro*	(157)	Denosumab (60 mg every 6 months for 24 months) did not slow the progression of AV calcification in patients with AS	(151)

### 10.1 Targeting macrophage polarization

Pro-inflammatory macrophages are traditionally seen as drivers of CAVD by promoting fibrosis and calcification, whereas immunomodulatory macrophages are thought to counteract disease by resolving inflammation, suggesting that therapeutic benefit could be gained by shifting polarization toward the latter phenotype.

Emerging evidence suggests that long non-coding RNAs (lncRNAs) regulate macrophage polarization (116, 117) and may contribute to AV calcification. Notably, AFAP1-AS1, a lncRNAs which promotes the osteogenic differentiation of VICs (118), is enriched in calcified AVs (119). Overexpression of AFAP1-AS1 in THP-1-derived macrophages suppressed the immunomodulatory profile and promoted a pro-inflammatory state, whose CM enhanced osteogenic marker expression, ALP activity, and calcification in VICs. Conversely, AFAP1-AS1 silencing favored an immunomodulatory phenotype, and the resulting CM suppressed VIC osteogenic transition and calcification. These findings suggest that AFAP1-AS1 drives AV calcification by skewing macrophage polarization toward a pro-inflammatory, pro-osteogenic state, making it a promising therapeutic target in CAVD.

In recent years, increasing attention has been given to the role of IL-37 in modulating macrophage polarization. In CAVD, an inverse relationship has been observed between IL-37 expression and pro-inflammatory macrophage polarization (120). *In vitro*, recombinant human IL-37 suppressed the expression of iNOS, CD11c, IL-6, and MCP-1 in pro-inflammatory macrophages, while enhancing CD206 and IL-10 in immunomodulatory macrophages. This effect was associated with inhibition of the NF-κB and Notch1 pathways. Beyond macrophages, IL-37 also suppressed osteogenic responses of human VICs *in vitro* and alleviated valve lesions in mice (121). Consistently, recombinant IL-37 reduced inflammation and prevented mineralization in human VICs (122, 123). Collectively, these findings suggest that IL-37 may represent a promising therapeutic target in CAVD by shifting both macrophages and VICs away from a pro-inflammatory state, thereby attenuating valvular inflammation and calcification.

In 2023, Salazar-Puerta et al. investigated an innovative approach to enhance the generation of immunomodulatory macrophages using engineered EVs as nanocarriers to deliver molecular factors that reduce inflammation (124). EVs were derived from human dermal fibroblasts and loaded with the myeloid transcription factors CEBPA and Spi1, which are known to induce the transdifferentiation of endothelial cells into macrophages. The authors demonstrated that these EVs effectively promoted the conversion of VECs in aortic valve tissue *ex vivo* into anti-inflammatory macrophage-like cells. These findings suggest that engineered EVs could serve as next-generation nanocarriers to reprogram VECs into anti-inflammatory macrophages, offering a promising therapeutic strategy to modulate macrophage polarization and potentially prevent AS.

While targeting macrophage polarization appears promising, several aspects should be considered. Notably, immunomodulatory macrophages secrete TGF-β, a potent driver of AV fibrosis, raising uncertainty about whether promoting this phenotype is truly a viable therapeutic strategy. To date, no study has directly investigated whether TGF-β secretion by immunomodulatory macrophages affects the fibrotic activity of VICs. Clarifying this issue is essential if macrophage polarization is to be therapeutically steered toward the immunomodulatory phenotype. Besides, macrophage polarization is highly dynamic and reversible, posing challenges for developing drugs with sufficiently long half-lives to maintain macrophages in a protective state. Finally, it remains unclear whether macrophage phenotypes can be selectively targeted without compromising their essential functions in innate immunity and host defense.

### 10.2 Targeting inflammation

In recent years, it has become clear that local inflammation, whether driven by macrophages or VICs, plays a major role in CAVD. This has led to the idea that targeting pro-inflammatory cytokines could represent a promising therapeutic strategy.

#### 10.2.1 Targeting Il6

Interleukin-6 has emerged as a genetic risk factor for AS (125). IL-6 expression is elevated in AV leaflets where it correlates with tissue remodelling (126, 127), and increased circulating IL-6 levels are strongly associated with HF and increased mortality in patients with AS (4). *In vitro*, IL-6 neutralization reduces osteogenic transition and calcification of human VICs (128). Notably, Mendelian randomization studies have linked genetically predicted treatment with tocilizumab (an antibody targeting the IL-6 receptor) to a reduced risk of AS, thereby supporting the rationale for IL-6 inhibition in this disease (129). Collectively, these findings suggest that IL-6 blockade may provide therapeutic benefits in CAVD. Antibodies targeting IL-6, such as ziltivekimab (130, 131) and clazakizumab (132), are currently being evaluated for their cardiovascular effects in patients with advanced chronic kidney disease (CKD) and persistent inflammation. Should these trials yield positive results, further studies will be needed to determine whether IL-6 inhibition can slow AS progression in patients with and without renal impairment.

#### 10.2.2 Targeting IL-8/CXCR2 axis

In patients, elevated circulating IL-8 levels are associated with faster AS progression (59). CXCR2, an IL-8 receptor, is upregulated in calcified regions of AV samples (59). *In vitro*, pharmacological inhibition of CXCR2 prevents ECM degradation and calcification in human VICs and in rats with CKD (59). Moreover, CXCR2 antagonism reduced monocytosis and macrophage infiltration in porcine AVs implanted subcutaneously in rats, which led to decreased valvular calcification (133). Collectively, these findings suggest that IL-8 blockade may hold therapeutic potential for preventing or slowing AS. Several IL-8 antagonists are currently under development, including neutralizing antibodies such as BMS-986253 (ividalimab), and small-molecule inhibitors of CXCR1/2 such as reparixin and navarixin. These agents are in phase 2 clinical trials for cancer, chronic obstructive pulmonary disease, and other inflammatory diseases, but have not yet been tested in AS or cardiovascular calcification.

#### 10.2.3 Targeting other cytokines

The literature suggests that, beyond IL-6 and IL-8, macrophage-derived cytokines such as IL-1β and TNF-α may influence valvular remodeling, making them potential targets to prevent AS.

Three IL-1-targeting agents are already clinically available: anakinra (a recombinant IL-1 receptor antagonist), rilonacept (a fusion protein targeting IL-1 receptors), and canakinumab (a monoclonal antibody against IL-1β). Although these drugs are not specifically approved for cardiovascular indications, they have been tested in patients with cardiovascular diseases (134). Notably, the CANTOS trial demonstrated that canakinumab reduced systemic inflammation and improved outcomes in post-myocardial infarction HF (135). Anakinra has also shown benefits on cardiorespiratory function and quality of life in HF, particularly in patients with preserved ejection fraction (136, 137). To date, none of these IL-1 inhibitors has been evaluated for preventing or treating AS.

Several TNF-α-targeting agents are FDA-approved and widely used in autoimmune diseases such as rheumatoid arthritis and Crohn’s disease. These include TNF-α receptor–Fc fusion proteins (etanercept), chimeric antibodies (infliximab), fully human antibodies (adalimumab, golimumab), and PEGylated humanized antibodies (certolizumab pegol) (138). However, to date, none of these therapies have been evaluated for the prevention or treatment of AS.

#### 10.2.4 Targeting the NLRP3 axis

The NLRP3 inflammasome plays a pivotal role in innate immunity by activating caspase-1, leading to the maturation and secretion of the pro-inflammatory cytokines IL-1β and IL-18. Emerging evidence suggests that targeting NLRP3 in macrophages could protect against valvular calcification (139). In ApoE−/− mice fed a high-fat diet for 24 weeks, treatment with the NLRP3 inhibitor CY-09 (2.5 mg/kg/day, intraperitoneally for 42 days) improved AV function and reduced both calcification and the valvular expression of osteogenic markers (140). In this model, NLRP3 inhibition prevented macrophage polarization toward a pro-inflammatory phenotype, lowering IL-6 and TNF-α levels, and reduced the ratio of pro-inflammatory (CD11c+) to immunomodulatory (CD206+) macrophages, although it did not significantly affect the absolute number of CD206+ macrophages infiltrating the valve. Consistent with these data, *in vitro* studies have shown that anti-inflammatory compounds can suppress NLRP3 activation in VICs, thereby reducing calcification (141). Although several specific oral NLRP3 inhibitors are currently in development, no clinical trials have yet assessed their potential to slow AS progression. In this context, triggering receptor expressed on myeloid cells 2 (TREM2), whose expression is elevated in CAVD samples, has recently emerged as a key regulator of macrophage NLRP3 inflammasome activation and the subsequent mineralization of VICs (142). Indeed, suppression of TREM2 has been shown to activate NLRP3, leading to increased secretion of IL-1β, TNF-α, and IL-6, along with elevated production of reactive oxygen species. In ApoE-/- mice fed a high-fat diet, TREM2 inhibition exacerbated AV calcification. Future studies should assess whether AL002a—a novel agonistic antibody targeting TREM2 (143)—can confer protective effects in preclinical models of CAVD. Whether colchicine, a non-specific NLRP3 inhibitor that reduces ischemic events following myocardial infarction (144), also exerts protective effects against CAVD remains to be investigated. Importantly, IL-18, whose maturation depends on NLRP3 activation, is implicated in both the myofibroblastic and osteogenic transition of VICs (58). Thus, the protective effects of NLRP3 inhibition observed in pre-clinical studies may, at least in part, result from reduced IL-18 activity. Future preclinical studies should explore whether neutralizing IL-18 using recombinant IL-18 binding proteins (145), blocking IL-18 with neutralizing antibodies (146), or inhibiting its signaling via IL-18 receptor antagonists (147) can prevent valvular remodeling.

#### 10.2.5 Key considerations for anti-cytokine therapies

Despite the well-established link between inflammation and AS, a search of ClinicalTrials.gov (accessed July 7, 2025) confirms that no trials are currently testing anti-cytokine or anti-inflammatory therapies for the prevention or treatment of AS. Yet these cytokines remain particularly attractive targets, especially since clinically approved agents against them are already available. Timing is a major challenge, as patients with established AS—already exhibiting fibro-calcific remodeling and osteogenic differentiation—are unlikely to benefit significantly, since inflammation may no longer drive disease progression. Earlier intervention, during subclinical stages or aortic sclerosis, may be more promising. However, the high cost of biologics, the large patient populations required, and the long follow-up periods pose considerable practical obstacles to conducting such trials. Nonetheless, millions of patients worldwide are treated annually with TNF-α inhibitors, predominantly for rheumatoid arthritis, and many others receive biologics targeting IL-1β and IL-6. Observational studies examining CAVD outcomes in these populations could provide valuable insights and help design adequately powered clinical trials with CAVD as a primary endpoint. Moreover, given the central role of NF-*κ*B in inflammation-driven CAVD, targeting this transcription factor or other inflammation-related pathways could expand therapeutic opportunities in this field.

### 10.3 Restoring pyrophosphate

Bisphosphonates—including ibandronate, alendronate, zoledronate, and etidronate—are PPi analogs widely prescribed to increase bone density and reduce fracture risk in patients with osteoporosis. In a preclinical study, zoledronic acid halted AV calcification induced by excessive vitamin D in New Zealand rabbit (148). To date, only few clinical studies have assessed the effects of bisphosphonates on AS progression and outcomes. In a retrospective pilot study from 2010, Sterbakova et al. found that bisphosphonate use was independently associated with slower progression of mild AS in patients with preserved kidney function (149). In 2020, Alishiri et al. reported that alendronate (70 mg weekly over 24 months) slowed stenosis progression and improved clinical outcomes in patients with AS and concurrent osteoporosis (150). By contrast, a more recent study by Pawade et al. found that alendronic acid (70 mg weekly for 24 months) did not significantly affect AV calcification progression in patients with established AS (151). Further research is needed to determine whether bisphosphonates can prevent or slow AS progression.

Recent research has shown that ALP inhibitors can block inflammatory activation, osteogenic transformation, and mineral deposition in VICs (152, 153), but their effects on AS *in vivo* remain unexplored.

### 10.4 Targeting RANKL

The RANKL/OPG pathway plays a key role in regulating AV calcification. RANKL is present in human CAVD but is absent in healthy valves. Conversely, osteoprotegerin (OPG), a decoy receptor that inhibits RANKL signalling, is reduced in areas of focal valvular calcification (106). Evidence indicates that RANKL promotes pro-inflammatory macrophage polarization (154). *In vitro*, RANKL stimulation of BMDMs increases IL-6 and TNF-α secretion, which in turn promotes VSMC calcification; this effect is blocked by neutralizing antibodies against IL-6 or TNF-α (155). Given the role of these cytokines in VIC differentiation and mineralization, similar mechanisms likely contribute to AV leaflet calcification. Notably, RANKL enhances matrix calcification, increases ALP activity, and activates RUNX2 in cultured VICs (106). In contrast, exogenous OPG reduces AV calcification in hypercholesterolemic LDLR-/- ApoB100/100 mice by inhibiting osteogenic differentiation, without affecting fibrosis or lipid accumulation (156). Denosumab, a monoclonal antibody clinically used to inhibit RANKL and prevent bone resorption, also suppresses VIC mineralization *in vitro* (157). However, Pawade et al. reported that denosumab (60 mg every 6 months for 24 months) did not slow the progression of AV calcification in patients with AS. In this study, no differences were observed in ^18^F-sodium fluoride uptake in the valve or in peak aortic jet velocity, indicating a lack of effect on calcification activity or hemodynamic progression (151). To our knowledge, this study—which included a small number of patients (*n* = 49 denosumab, *n* = 50 placebo) and a predominance of male participants—remains the only clinical evaluation of RANKL inhibition in AS. Further research is needed to assess whether denosumab could be an effective strategy to prevent or slow AS progression.

## 11 Macrophages and structural valve deterioration (SVD)

Emerging evidence suggests that macrophage activity contributes to structural valve deterioration (SVD), a progressive degeneration of bioprosthetic AVs characterized by tissue changes such as calcification, stiffening, or tears, which can lead to stenosis or regurgitation and limit long-term durability (158). SVD affects about 30% of patients within a median of 6 years post-AVR, with most cases (∼80%) arising after 10 years. This condition doubles the risk of mortality and often necessitates valve reintervention within 10–15 years of the initial surgery (159).

Glutaraldehyde fixation of bioprosthetic tissues, used to stabilize the ECM prior to implantation, induces compositional changes that promote monocyte recruitment and macrophage activation (160, 161). Despite efforts to reduce immunogenicity, xenografts retain carbohydrate antigens like αGal and Neu5Gc, which can activate complement pathways and further enhance immune cell infiltration (161, 162). Consequently, immune cells—including macrophages, foam cells, and multinucleated giant cells—are observed in histological samples of degenerated bioprosthetic AVs, often colocalizing with lipid deposits, mirroring patterns observed in native AS (163–165). Elevated MMP activity in macrophage-rich regions supports their role in ECM remodelling (163, 164). This immune activation also triggers pro-thrombotic processes through platelet adhesion and coagulation. Subclinical leaflet thrombosis, affecting up to 30% of patients within the first year post-implantation, increases SVD risk (166, 167). Fibrin deposits further enhance monocyte adhesion and macrophage activation (168). In animal models, fibrin deposition has been linked to calcification in bovine pericardium (165, 169). Mechanical stressors—such as hypertension, small aortic annulus, or patient-prosthesis mismatch—likely exacerbate SVD via macrophage activation (160). Together, these findings indicate that persistent or reactivated monocyte/macrophage-driven inflammation plays a central role in bioprosthetic valve degeneration. Given that free aldehyde groups resulting from glutaraldehyde fixation, along with phospholipids and circulating calcium ions, promote passive ECM calcification (170), it is plausible that this passive Ca/P deposition further drives pro-inflammatory macrophage polarization. However, to our knowledge, no studies have directly assessed macrophage polarization within bioprosthetic valves affected by SVD, highlighting a critical gap for future research.

## 12 Conclusion

Over the past decade, studies have highlighted the pivotal yet dualistic role of macrophages in AV remodeling. Pro-inflammatory macrophages are now well established as drivers of VIC osteogenic differentiation and mineralization, mediated by the secretion of pro-inflammatory cytokines and EVs enriched with small RNAs. Immunomodulatory macrophages, long considered protective due to their IL-10–mediated resolution of inflammation, are now thought to also promote myofibroblastic differentiation of VICs and VECs. Given the potentially deleterious effects of both macrophage subtypes, strategies aimed solely at shifting polarization toward an immunomodulatory phenotype should be approached with caution. Recent preclinical evidence suggests that broader modulation of inflammation, rather than targeting a single phenotype, may be a more effective therapeutic strategy. However, despite the availability of therapeutic agents targeting key pro-inflammatory cytokines such as IL-1β, IL-6, and TNF-α, no studies have yet specifically evaluated the impact of anti-interleukin therapies on CAVD. Observational studies in patients receiving these therapies for other indications could provide valuable insights and guide the design of adequately powered clinical trials with CAVD as a primary endpoint.

An important consideration in AS research is the influence of comorbid conditions on VIC and macrophage physiology, as these factors can significantly influence disease progression. Chronic kidney disease provides a striking example, as AV remodeling occurs more frequently, earlier, and progresses faster in CKD patients, leading to a nearly threefold increase in mortality compared with the general population (171–173). In CKD, the accumulation of uremic toxins promotes pro-inflammatory macrophage activation while impairing their ability to adopt an osteoclast-like phenotype (174–176), thereby fueling valvular calcification. Bicuspid aortic valve (BAV) disease represents another context in which the crucial role of macrophage involvement remains underappreciated. Macrophage density and distribution are higher and more widespread in BAV compared to tricuspid AVs (177–179), with elevated expression of pro-inflammatory markers such as iNOS, phosphorylated-p65, and TNF-α, and reduced levels of immunomodulatory markers including CD163, Arg-1, and IL-10 (178, 180). Notably, macrophages expressing neopterin—a by-product of the guanosine triphosphate pathway that amplifies oxidative stress—are more abundant in BAV patients (179). Whether this pro-inflammatory macrophage profile contributes to the higher calcification propensity observed in BAV remains unclear. Finally, given that macrophage-derived cytokines can differentially influence VIC phenotype depending on sex (56), future studies should systematically account for sex as a biological variable. Macrophages from male and female donors may differ in activation states, cytokine profiles, and interactions with VICs, potentially influencing CAVD progression and therapeutic responses.

Given the central role of macrophages in regulating valvular remodeling, it is reasonable to consider their potential as biomarkers predictive of AS progression or outcomes. Direct assessment of macrophages as biomarkers is challenging due to their localization within the valvular tissue; however, circulating factors that reflect macrophage activity may offer a practical alternative. Supporting this approach, Mueller et al. showed that circulating levels of macrophage migration inhibitory factor (MIF) predict rapid AS progression, with MIF-associated biomarkers strongly linked to an accelerated disease course (181). Considering the crucial role of macrophage subsets in CAVD, future studies should investigate how biomarkers of macrophage activity—such as MIF, IL-8, IL-6, TNF-α, and IL-1β—could help identify patients at high risk of rapid progression and how these markers might be integrated with perioperative risk scores to select AS patients most likely to benefit from AVR.
